# Genome Sequences of Five Indian Canine Rabies Virus Isolates Obtained Using Oxford Nanopore Technologies Sequencing

**DOI:** 10.1128/mra.01246-21

**Published:** 2022-04-26

**Authors:** Ankeet Kumar, Sujith S. Nath, Ashwin Sridhar Sudarshan, Vinithra Iyer, Niti B. Jadeja, Neha Panchamia, Indrakshi Banerji, Abi Tamim Vanak, Utpal Tatu

**Affiliations:** a Department of Biochemistry, Indian Institute of Science, Bangalore, Karnataka, India; b Ashoka Trust for Research in Ecology and the Environment, Bangalore, Karnataka, India; c RESQ Charitable Trust, Pune, Maharashtra, India; d DBT/Wellcome Trust India Alliance, Hyderabad, Telangana, India; e School of Life Sciences, University of KwaZulu-Natal, Durban, South Africa; Portland State University

## Abstract

We report five canine rabies virus genome sequences from India that were obtained from brain samples using Oxford Nanopore Technologies sequencing. The sequences will facilitate understanding of the evolution and transmission of rabies.

## ANNOUNCEMENT

Rabies is one of the oldest known viral diseases; it is caused by a virus belonging to the family *Rhabdoviridae* and genus *Lyssavirus* that infects humans as well as animals. In India, free-ranging dogs are the most common carrier of this virus (1).

Rabies virus (RABV) has a 12-kb negative-sense RNA genome encoding five proteins, i.e., nucleoprotein, phosphoprotein, matrix protein, glycoprotein, and RNA-dependent RNA polymerase (2). To determine the whole-genome sequences of RABV found in rabid dogs, ~1-g brain samples were collected from 374 dogs in Pune, India, in 2018. The samples were tested for rabies antigen using the lateral flow assay test (Bionote, Hwaseong-si, South Korea) and rabies genes using reverse transcription (RT)-PCR (3). Of 203 positive samples, 5 samples were subjected to RNA isolation using a Zymo Research kit (R1054; New England Biolabs) following the manufacturer’s protocol with DNase treatment, and concentrations were measured using a Qubit fluorometer (Invitrogen). cDNA was prepared using random hexamers from the LunaScript kit (E3010L; New England Biolabs) followed by multiplex tiled PCR with Q5 Hot Start high-fidelity DNA polymerase (M0493S; New England Biolabs) using custom-designed overlapping primers to amplify 400-bp regions across the genome. Primers (https://figshare.com/articles/media/Genome_sequences_of_Indian_canine_Rabies_virus_using_Oxford_Nanopore_Technology/17694347?file=32385851) were designed using the NNV-RAB-H strain with approximately 90 to 100 bp of overlap. Multiplex tiled PCR was performed in two batches, to avoid enrichment of 90- to 100-bp overlapping segments (4). The quality of 400-bp amplicons was confirmed by agarose gel electrophoresis. Library preparation was performed with the ligation-based kit (SQK-LSK109) from Oxford Nanopore Technologies (ONT). The barcode expansion kit (EXP-NBD104) from ONT was used to barcode the five samples. Sequencing was performed on the MinION system, and data collected for each sample can be found in Table 1. Gaps were seen in the genomes and were filled by a second round of sequencing using the same primers for the gap regions (https://figshare.com/articles/dataset/Primers_used_for_gaps_xlsx/19402688).

**TABLE 1:** Sequencing data for the isolates and comparison with the closest relative

Sample name	SRA accession no.	GenBank accession no.	Total no. of sequences	Total length (bp)	GC content (%)	Identity to closest relative (%)	GenBank accession no. for closest relative
Pune_RABV1	SRX13297356	OL422906.1	309,790	157,638,492	45.71	97.84	LT909541.1
Pune_RABV2	SRX13297357	OL422907.1	371,403	185,915,457	45.53	97.38	LT909541.1
Pune_RABV3	SRX13297358	OL422908.1	320,692	161,980,243	45.71	97.46	LT909541.1
Pune_RABV4	SRX13297359	OL422909.1	311,508	173,133,768	45.56	97.53	LT909541.1
Pune_RABV5	SRX13297360	OL422910.1	166,835	98,505,425	45.71	97.61	LT909541.1

Read base calling was performed using Guppy v3.5.2 for the first round of sequencing and Guppy v5.0.7 for the second round (5). Reads with quality scores above 7 were used and demultiplexed using Guppy to obtain the reads for each of the five samples. The consensus sequence was obtained by using the commands from the file minion.py from the ARTIC protocol (https://github.com/artic-network/fieldbioinformatics/blob/master/artic/minion.py) (6), and the parameters used were as specified in the protocol. Reads 200 to 700 bp long were mapped against the reference genome (strain NNV-RAB-H [GenBank accession number EF437215.1]) (normalized to 150× coverage) using minimap2 v2.17, and the option –x map-ont was used to map ONT reads to the reference. The output SAM files were indexed and sorted for further steps. Variant calling was performed using medaka v1.0.3 (https://github.com/nanoporetech/medaka) as a part of the ARTIC pipeline, and final consensus sequences were obtained. Medaka was run separately for the reads from the first and second rounds to account for the different models needed due to the different Guppy versions and different versions of the flow cells used (r941_min_high_g351 for the initial batch of reads and r103_min_high_g360 for the gaps sequenced in the second run). The VCF files were merged by sample and category using bcftools merge. Only variants identified in either set of sequencing reads with coverage of ≥20× and nonframeshift mutations were considered for building the consensus sequence.

The GC contents ranged from 45.53 to 45.71% among the five genomes. Information related to the data collected, closest relative genome, and percentage of identity to the closest relative is available in Table 1. Identity among our genomes ranged from 94.14 to 99.4%.

Permissions for animal sampling were granted by concerned authorities of the Ashoka Trust for Research in Ecology and the Environment (ATREE) and the Indian Institute of Science under approval numbers AAEC/101/2016 and CAF/Ethics/831/2021, respectively.

### Data availability.

The raw reads and the genome sequences were submitted to NCBI with SRA accession numbers SRX13297356, SRX13297357, SRX13297358, SRX13297359, and SRX13297360 and GenBank accession numbers OL422906.1, OL422907.1, OL422908.1, OL422909.1, and OL422910.1. The primers used and the coverage of genes are reported on figshare (https://figshare.com/articles/media/Genome_sequences_of_Indian_canine_Rabies_virus_using_Oxford_Nanopore_Technology/17694347?file=32385851 and https://figshare.com/articles/media/Genome_sequences_of_Indian_canine_Rabies_virus_using_Oxford_Nanopore_Technology/17694347?file=32385860, respectively). NNV-RAB-H (GenBank accession number EF437215.1) was used as the reference genome for mapping and primer design.
